# Report of 15 injuries caused by lionfish (*pterois volitans*) in aquarists in Brazil: a critical assessment of the severity of envenomations

**DOI:** 10.1186/s40409-015-0007-x

**Published:** 2015-03-20

**Authors:** Vidal Haddad, Hamilton Ometto Stolf, José Yamin Risk, Francisco OS França, João Luiz Costa Cardoso

**Affiliations:** Departament of Dermatology and Radiotherapy, Botucatu Medical School, Univ Estadual Paulista (UNESP), Caixa Postal 557, Botucatu, São Paulo State 18618-970 Brasil; Vital Brazil Hospital, Butantan Institute, São Paulo, São Paulo State Brazil; Department of Infectious and Parasitic Diseases, School of Medicine, University of São Paulo (USP), São Paulo, São Paulo State Brazil; Private Clinic, Ubatuba, São Paulo State Brazil

**Keywords:** *Pterois volitans*, *Pterois miles*, Venomous fish, Animals invasions, Aquarism, Lionfish, Envenomations

## Abstract

Lionfish are venomous fish that belong to the Scorpaenidae family. Individuals of this family and those of the Synanceiidae family comprise most of the existing venomous fish in the world. Lionfish are originally found in the Indo-Pacific, but they have received special attention in the last years for their dissemination in the Atlantic Ocean, with the emergence of large populations in the USA, Caribbean and South America. Because of its beauty, this fish has always been present in private and commercial aquariums around the world. Herein, we describe 15 envenomations in aquarists registered in a period of eighteen years (1997–2014). The stings caused excruciating pain and marked inflammation, with local erythema, edema, heat, paleness and cyanosis. In one case, it was possible to observe vesicles and blisters. There were no skin necroses or marked systemic manifestations. We discuss the possible coming of the fish to South America and the circumstances and clinical impact of the envenomations.

## Background

Lionfish are venomous fish belonging to the Scorpaenidae family. These animals and those of the Synanceiidae family (*Synanceia* genus - stonefish - and others) comprise most of the existing venomous fish. Envenomations provoked by stonefish are very serious and potentially fatal since this fish is considered the most venomous throughout the globe. However, scorpionfish and lionfish can also cause significant envenomation due to the systemic action of their venoms.

Members of the Scorpaenidae family can be divided into two groups regarding their importance to human health: lionfish (*Pterois* genus and others) and scorpionfish (*Scorpaena* genus and others). The envenomation caused by scorpionfish is severer than that caused by lionfish [1-4].

**Figure 1:**
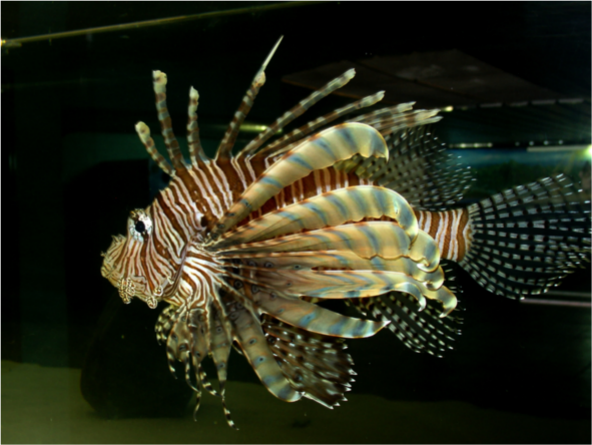
**Lionfish (** ***Pterois volitans*** **).** Note the exposed sharp point of the ray on the dorsal fin. Photo by: Vidal Haddad Junior.

Lionfish present long and slender spines (bony rays in the fins) (Figure 1). The rays are covered by an epithelial sheath that contains venom-producing glands in the grooves of upper two-thirds of the spine. The venom flows to the wound when the ray of the fin penetrates the skin of the victim and the epithelium is broken. There are 12 to 13 rays or spines in the dorsal fin, two in the pelvic fin and three in the anal fin. The pectoral spines do not have venom [5,6].

Lionfish are originally inhabitants of tropical waters of the Indo-Pacific region. In other places, due to their beauty, they are common aquarium pets. The majority of the reported envenomations by lionfish occur in the upper extremities (hands) of individuals that were handling the fish in aquariums. The main symptom is the excruciating local pain, which may radiate throughout the root of the affected limb. The pain increases in 1 to 2 hours and typically persists for 6 to 12 hours [7-13]. Moreover, the painful process may last for weeks. There is marked inflammation, which causes important erythema, edema and local heat. In some cases, it is possible to observe local cyanosis, paleness, vesicles and blisters. Rarely, the sting site presents skin necrosis [7-13].

Lionfish venom may provoke systemic manifestations such as cardiac effects and affect blood pressure. These results are thought to be due to nitric oxide release [14,15]. In humans, *Pterois* venom usually causes nausea, vomiting, cold sweating, fever, dyspnea, convulsions, abdominal pain and syncope. Paralysis of the limbs and cardiac failure are infrequently observed. To the best of our knowledge, there are no published reports of death, since the venom is probably not lethal to healthy humans [16]. The development of anaphylaxis and severe infections are always possible and require immediate emergency medical treatment [14,15].

There is a classification of the severity of the envenomation caused by lionfish in three degrees of local effects, whereas degree I shows only erythema and edema (most injuries), degree II also presents vesicles and blisters and degree III is characterized by skin necrosis [7-13].

Lionfish has received special attention in recent years for its dissemination in the Atlantic Ocean, with emergence of large populations in the USA (from Florida to Cape Hatteras, North Carolina), in several Caribbean countries (Bermuda, Bahamas, Cuba, Dominican Republic, Jamaica, Puerto Rico, Turks and Caicos, Cayman Islands, Belize, Haiti, U.S. Virgin Islands, Mexico, Aruba, Curaçao, and Bonaire) and more recently in South America (Colombia and Venezuela) [5]. Their environmental impact is catastrophic, due to the predatory feeding habits and their capacity of expansion. Lionfish feed on crustaceans and economically important reef fish, which brings a significant impact to the affected sites.

Additionally, they do not have predators in invaded areas and their reproduction is fast, favoring their uncontrolled dissemination. Once they were already established in the USA and the Caribbean, they became one of the most common predators of reef areas. Additionally, these fish have currently been observed in South America, with possibility of expansion to the rest of the continent in its portion bounded by the Atlantic Ocean, including the large Brazilian coast [17-19].

On the invasion of Indo-Pacific Lionfish in Brazilian waters, recent research indicates that a combination of the effects of the Amazon/Orinoco river plumes and prevailing currents along northern Brazil may slow the pace of the potential invasion, which could help eradication programs if action is taken before lionfish become widespread and established in Brazil [20]. More recently, researchers have captured a specimen of lionfish (*Pterois volitans*) off the southeastern Brazilian coast, in the Rio de Janeiro state. The collected fish was genetically linked to the Caribbean population and the consequences of this first capture have yet to be evaluated [21].

The aim of the present communication is to report injuries in 15 aquarists from São Paulo State, Brazil, and to evaluate the clinical manifestations caused by the envenomation regarding the severity and risks for patients injured by lionfish.

## Case presentation

**Figure 2:**
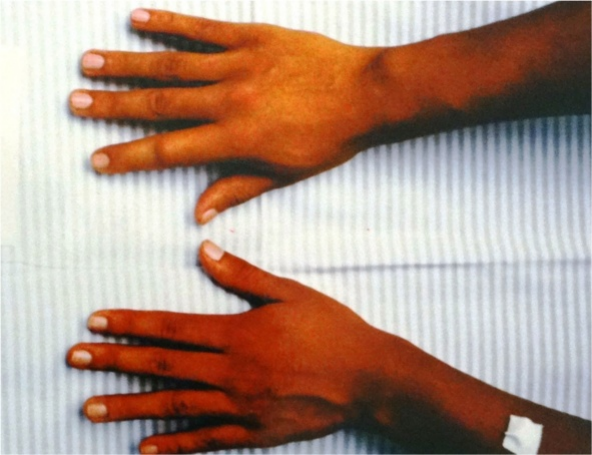
**Envenomation that caused initial intense pain on the right index finger of an aquarist, observed ten hours after the injury.** Photo by: Francisco O. S. França.

**Figure 3:**
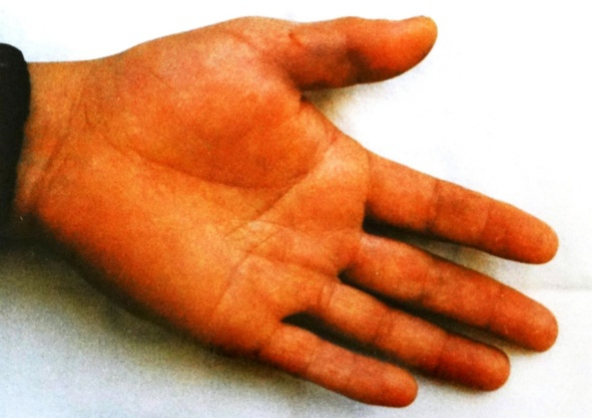
**Puncture on the left thumb with mild edema and cyanosis two hours after the sting.** The pain was intense. Photo by: João Luiz Costa Cardoso.

**Figure 4:**
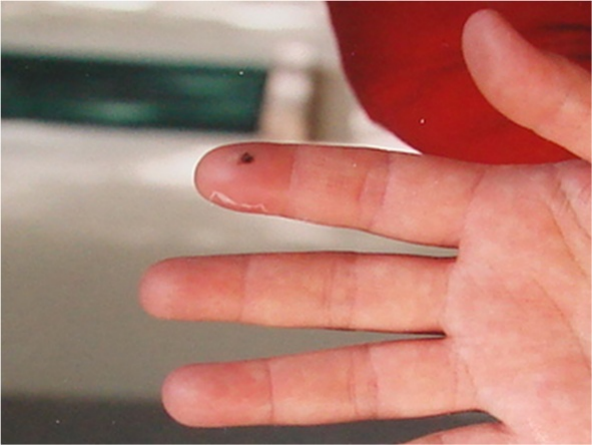
**In this injury in the middle finger of the right hand of an aquarist, the pain was the initial symptom.** Three hours after, the sting site showed important edema and erythema. Photo by: José Yamin Risk.

**Figure 5:**
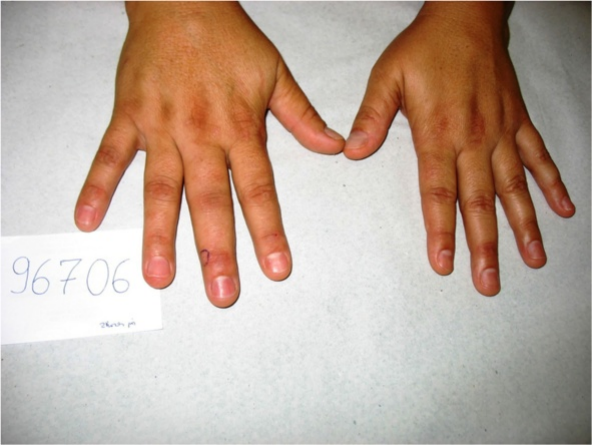
**There were edema and erythema on the index and medium fingers of the right hand of an aquarist.** The sting was on the index finger. Photo by: Vidal Haddad Junior.

**Figure 6:**
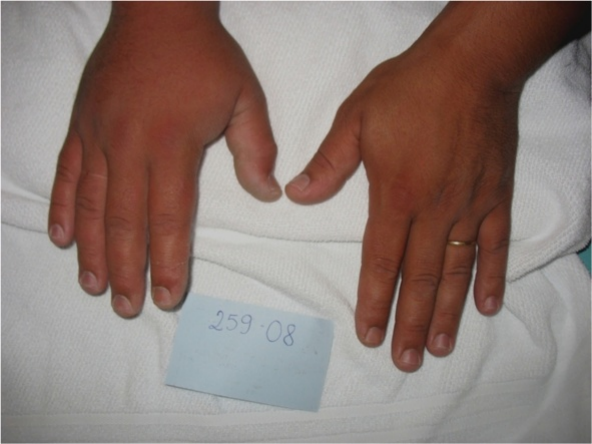
**Edema and erythema present in the right hand of a victim that suffered a puncture in the middle finger two hours after the exam.** Photo by: João Luiz Costa Cardoso.

Fifteen envenomations were registered in a period of eighteen years (1997–2014) in the Vital Brazil Hospital of the Butantan Institute and the Department of Dermatology of the Botucatu Medical School, at the São Paulo State University, Brazil (Figures 2, 3, 4, 5 and 6). The patients were observed and treated by the authors and had their cases described in previous studies [10,11] in accordance with the ethical committees of both the institutions (Table 1).

**Table 1:** **Clinical manifestations presented by the victims described in the study**

	**Intense**	**Moderate**	**Discrete**	**Total**
Erythema	12 (80.00%)	3 (20.00%)	–	15 (100%)
Edema	15 (100%)	–	–	15 (100%)
Local heat	15 (100%)	–	–	15 (100%)
Pain	14 (93.33%)	1 (6.6%)	–	15 (100%)
Blisters	–	1 (6.6%)	–	15 (100%)
Skin necrosis	–	–	–	15 (100%)
Nausea/vomit	–	2 (13.33%)	–	15 (100%)
Dyspnea	–	–	–	15 (100%)
Fever	–	2 (13.33%)	–	15 (100%)
Muscular weakness	–	–	–	15 (100%)

All the wounds were caused by specimens of *Pterois volitans* and the patients were aquarists. The injuries did occur when the victims were handling fish or cleaning the aquarium. Rays of the dorsal fin of the fish provoked lesions that affected the hands (five victims) or fingers (ten victims, eight wounds on the index finger). The initial and most marked symptom was intense pain referred as excruciating by the victims. The sting sites (hands and fingers) showed important inflammation, whereas edema and local heat were observed in all patients. Intense erythema and pain were reported by 12 and 14 victims, respectively. Pain was classified as intense when the patient presented behavioral changes, cold sweating and restlessness. Although sphincter relaxation, fetal position and aggressiveness may be observed, these symptoms were not observed in the present cases.

Local paleness (13 patients) and cyanosis (six patients) were detected. The local effects of the venom were significant, but only one patient developed blisters in moderate intensity. Systemic effects were not observed, except for nausea and vomit, which can also be credited to the intense pain and the stress of the patients.

The pain usually decreased with the immersion of the affected region in hot water, but it would return if it was removed. Inflammation was not influenced by the use of hot water or anti-inflammatory drugs. In two patients, there were bacterial infections with expansion of erythema and additional area of edema and fever. They were treated with cephalexin 500 mg (orally) four times a day and healed without complications.

## Conclusions

The presence of lionfish (the red lionfish *Pterois volitans* and the devil firefish *Pterois miles*) in the South America brought some new facts that enrich the discussion about the problem. In fact, the spread of these fish (mainly *Pterois volitans*) was expected since the observation of the first specimens, spotted in the state of North Carolina (Atlantic coast of USA), on 1985 [4]. Due to the above-mentioned reasons (lack of predators, voracious feed on fish and crustaceans), these beautiful and dangerous fish have become common in areas far from their natural habitat, with the expected risks posed to the environment by a competent and highly skilled attacker.

Since five years ago, with the help of fishermen communities near the mouth of the Amazon River, we have monitored the presence of lionfish in Brazilian waters. To date, there are no reports of sighting or capturing the fish. This picture reinforces the theory of barrier estuary, but does not exclude the possibility of future invasions (although the likelihood of these is smaller, especially when we think how quickly the fish colonized the Caribbean region) [1-4].

The danger posed by the rays of the fins and their venomous glandular tissue (especially the dorsal fin rays) is real, but unusual. Envenomations caused by lionfish only happen in divers and fishermen, with no risk to bathers. Even in areas where fish are common, the envenomations are rare, since their characteristic appearance is important in the recognition and prevention of injuries.

We registered intense pain, edema, erythema and, in one case, blisters, but the injuries did not pose risks of cardiovascular failure or death in our patients [12,13]. The most important symptoms were the local inflammation and severe pain. Some signs and symptoms frequently observed in lionfish envenomation were not seen in our patients, since only one had blisters on the sting site and we did not register necrosis or systemic signs that could not be credited to stress caused by the pain [7-11].

Lionfish envenomation has always been present in treatment protocols in Brazil, even before the spread into the Atlantic Ocean. Treatment recommends immersion of the affected site (which is usually a finger of the hand) in tolerable hot water for 30–90 minutes, which helps to control the pain. The venoms of some fish may provoke important vasoconstriction, which explains the pain, pallor, cyanosis and skin necrosis at the sting site. The immersion in hot water provokes vasodilatation in the vessels, which overrides these effects. The most logical explanation for the significant effect of immersion in hot water for pain relief is not simply the thermolability of the venom, since the pain returns when the affected limb is removed from the hot water [3,4,22-24]. The secondary infections are always a possibility. Perforating wounds are always more likely to present infections, by deep inoculation of fungal and bacterial agents.

The manifestations caused by lionfish were mostly local and the severity of injuries was moderate, far less intense than those caused by other Scorpaenidae including scorpionfish (*Scorpaena*) or the deadly stonefish (*Synanceia*) of the Indo Pacific that can cause deaths by envenomation. In our view, accidents involving lionfish should have their severity classified by presence or absence of systemic phenomena, which, in theory, may threaten the life of the victim.

Given that venom of Scorpaenidae family has systemic action, the absence of systemic symptoms in our patients may indicate the inoculation of a small amount of toxins (the maximum number of perforations were three), or mild to moderate systemic effects on humans. The pain and inflammation were significant; therefore, awareness campaigns, first aid measures and prevention have to be carried out in risky areas. Nevertheless, this type of envenomation is as harmful as that caused by scorpionfish, marine stingrays and marine catfish, so the same care measures should be adopted [1-3,22-24]. Lionfish do not cause injuries to bathers and only affects a few populations (divers, fishermen) [25]. Certainly, the impact on the environment caused by these fish is huge and should be properly managed because profound changes may occur due to the increasing spread of the species *Pterois volitans*. Campaigns in the USA, the Caribbean and South America are extremely valid and must continue, but the hysteria caused by the fact that lionfish are venomous, however, is not justified.

## Consent

Written informed consent was obtained from patients for publication of this case report and any accompanying images.
